# Simple renal cysts are associated with ischemic stroke in young adults: a case–control study

**DOI:** 10.3389/fneur.2026.1926737

**Published:** 2026-10-05

**Authors:** Fangyi Li, Qingxi Zhang, Guohua He, Yangping Tong, Kangping Song

**Affiliations:** Department of Neurology, The Affiliated Changsha Central Hospital, Hengyang Medical School, University of South China, Changsha, Hunan, China

**Keywords:** case–control study, public health, risk factor, simple renal cyst, young stroke

## Abstract

**Background:**

The incidence of ischemic stroke among young patients is increasing, and ischemic stroke has become a major cause of death and disability in young adults. The key to alleviating this problem is to identify the risk factors and causes of stroke to optimize treatment. The current study aims to assess whether simple renal cysts are associated with the occurrence of ischemic stroke among young adults.

**Methods:**

Data from patients with acute ischemic stroke aged 18–50 years and age- and sex-matched controls who underwent medical examinations at the Affiliated Changsha Central Hospital, Hengyang Medical School, University of South China, from October 2022 to September 2025 were retrospectively collected. Potential risk factors (including blood pressure, fasting blood glucose, dyslipidemia, homocysteine, and simple renal cysts) were compared between young patients with stroke and controls. Binary logistic regression analysis was conducted to identify risk factors for ischemic stroke among young patients.

**Results:**

A total of 219 young patients with acute ischemic stroke and 219 controls were enrolled for analysis. Patients with elevated blood pressure, elevated blood glucose, and concurrent simple renal cysts had a higher risk of ischemic stroke. Simple renal cysts were associated with a higher risk of ischemic stroke in young adults, but they did not correlate with greater stroke severity.

**Conclusion:**

Simple renal cysts are associated with ischemic stroke in young adults and thus merit clinical awareness. Prospective multicenter cohort studies are expected to confirm this association and clarify potential mechanisms.

## 1 Introduction

Stroke is the second leading cause of death worldwide (1), and approximately 10–15% of all strokes occur in young people aged 18–50 years (2, 3). It is estimated that morbidity has increased by more than 40% over the past decades, as approximately 2 million young adults experience stroke every year globally (4). With the increasing incidence, stroke has become the eighth leading cause of death and the main cause of disability in American adults aged 25–45 years (5). Notably, stroke in young adults places a substantial burden on society and families compared with stroke in older adults, as it often leaves them disabled during their most productive years. Therefore, it is extremely important to reduce the morbidity, mortality, and disability associated with stroke in young adults. The key to addressing this issue is to identify the risk factors and causes of stroke to optimize treatment.

Epidemiological data suggest that most young adults with stroke have traditional risk factors for cerebrovascular disease, such as hypertension, dyslipidemia, diabetes, smoking, excessive alcohol consumption, and cardiovascular disease (6, 7). Carotid artery dissection is also one of the main causes of stroke in young adults (8). In a recent report, Deb et al. identified chronic kidney disease, cancer, sleep apnea, and bipolar disorder as additional risk factors for stroke among young populations (9). However, there are still a considerable number of young patients with stroke of unclear etiology, indicating that other potential risk factors are involved in the development of stroke in young adults. Sung et al. reported that the risk of stroke in patients with autosomal dominant polycystic kidney disease (ADPKD) was higher than that in patients without ADPKD in a large sample of one million individuals from the general population (10). Combined renin-angiotensin-aldosterone system (RAAS) blockade and statin therapy are effective strategies for controlling this increased risk. A recent population-based cohort study reported that polycystic kidney disease was independently associated with an increased risk of all stroke subtypes over a 15-year follow-up, with ischemic stroke having the highest incidence (11). Interestingly, as neurologists, we have found that young patients with stroke often have concurrent simple renal cysts in our clinical practice. However, few studies have explored the relationship between simple renal cysts and stroke in young adults. Population-based survey data indicate that simple renal cysts occur with appreciable frequency in young adults, with a prevalence of approximately 4.7% in those aged <40 years, yet their clinical significance is often underestimated (12). Accordingly, we conducted the current study to assess whether renal cysts are associated with the occurrence of ischemic stroke among young adults, which may contribute to stroke prevention strategies in this population.

## 2 Materials and methods

### 2.1 Study population

This was a retrospective population-based case–control study. We reviewed all patients with acute ischemic stroke (AIS) who were aged 18–50 years and admitted to the Affiliated Changsha Central Hospital, Hengyang Medical School, University of South China, from October 2022 to September 2025. A total of 219 patients were enrolled based on the following inclusion criteria: (1) were diagnosed with AIS using computed tomography (CT) or magnetic resonance imaging (MRI) according to the 2018 Chinese guidelines for the diagnosis and treatment of acute ischemic stroke; (2) aged 18–50 years (13); (3) underwent renal ultrasound examination; and (4) had complete records of clinical data, including the National Institutes of Health Stroke Scale (NIHSS) score, creatinine, fasting blood glucose (FBG), low-density lipoprotein (LDL), high-density lipoprotein (HDL), and triglycerides. In the present study, patients were excluded if they (1) were <18 or >50 years old; (2) lacked renal ultrasound results; (3) were diagnosed with ADPKD according to the published diagnostic criteria (14); or (4) had incomplete clinical data. The detailed patient enrollment workflow is shown in Figure 1. Then, 219 individuals matched for age and sex were selected as controls from the medical examination system of the Affiliated Changsha Central Hospital, Hengyang Medical School, University of South China, during the same period; these individuals had no history of stroke. The specific procedures were as follows: frequency matching was used, with the age distribution of the case group (stratified at 5-year intervals) serving as the sampling frame. All eligible individuals undergoing medical examinations were stratified by sex and age. Within each stratum, a computer-generated random number table was used to randomly select the same number of individuals as in the corresponding stratum of the case group. After enrollment, the age and sex distributions of the two groups were compared to ensure balance (*p* > 0.05).

**Figure 1:**
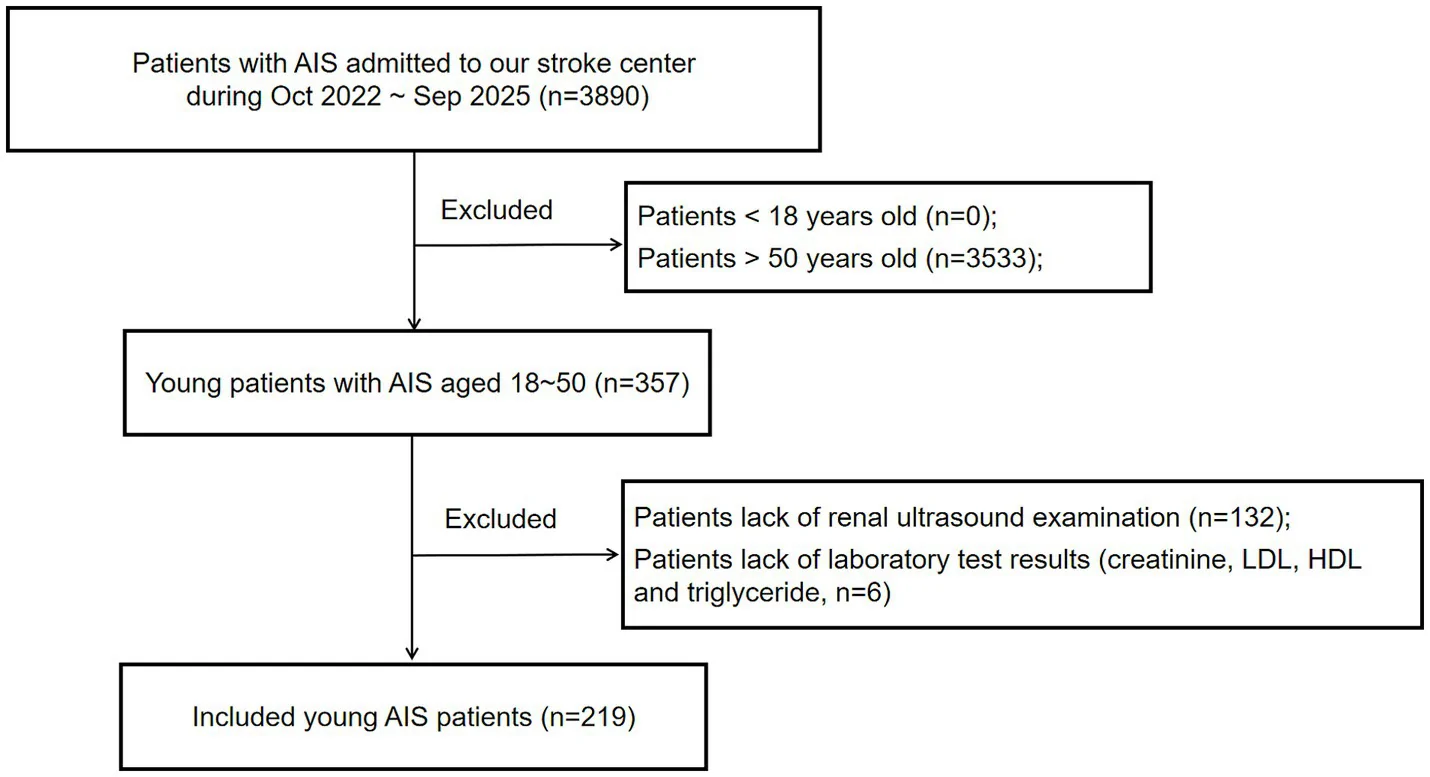
The flowchart of patient screening. AIS, acute ischemic stroke; HDL, high-density lipoprotein; LDL, low-density lipoprotein.

### 2.2 Data collection

Patients’ baseline data were collected and recorded by experienced neurologists. Clinical information included demographic characteristics (age and sex), medical history (diabetes mellitus, hypertension, hyperlipidemia, stroke, transient ischemic attack [TIA], coronary artery disease, and atrial fibrillation), and prehospital medications (antiplatelet agents, anticoagulants, statins, hypoglycemic treatments, and antihypertensive therapy). Physical examinations were performed immediately after admission and included measurements of body mass index (BMI), systolic blood pressure (SBP), diastolic blood pressure (DBP), and the NIHSS score. Blood samples were collected from all patients after fasting for at least 8 h for routine biochemical examinations, including hepatic function, creatinine, FBG, homocysteine, LDL, HDL, and triglycerides (measured using an automatic analyzer, HITACHI 7600, Japan). The aforementioned information was collected as baseline clinical characteristics rather than long-term prestroke risk profiles. Renal cysts were detected using color Doppler ultrasound (Aplio 400, Siemens 2000, Resona R9s, Germany) by experienced ultrasound physicians. Simple renal cysts were diagnosed by ultrasonography based on the following established criteria: (1) anechoic content without internal echoes; (2) posterior acoustic enhancement; (3) round or oval shape; and (4) sharp, thin, smooth posterior walls with well-demarcated borders (15). For the control group, corresponding clinical information was recorded except for medical history and prehospital medication use, as this information was unavailable. All ultrasound examinations were performed and interpreted by experienced ultrasound physicians. The diagnosis of simple renal cysts was made prospectively during routine clinical care, before assignment of case or control status for this study. Thus, the ultrasound physicians were blinded to the patients’ case–control status and clinical outcomes.

### 2.3 Patient and public involvement

Patients and/or the public were not involved in the design, conduct, reporting, or dissemination plans of this research. Data from the enrolled patients were extracted from available case files after approval from the Institutional Ethics Committee.

### 2.4 Statistical analysis

Statistical analysis was performed using SPSS software (IBM SPSS Statistics for Windows, version 26.0). To determine differences between patients with AIS and controls, continuous variables were presented as the mean ± standard deviation (SD) and analyzed using Student’s *t-*test if normally distributed; continuous variables that were not normally distributed were presented as the median (interquartile range [IQR]) and analyzed using the Mann–Whitney U test. Unordered categorical variables were presented as numbers (percentages), and chi-square tests or Fisher’s exact test were used for analysis, as appropriate. Binary logistic regression analysis was performed to determine independent risk factors for AIS. Specifically, all covariates with a *p-*value < 0.10 in the univariate analysis were considered candidates for the multivariable model. Multicollinearity among independent variables was assessed using the variance inflation factor (VIF), with VIF > 5 considered indicative of significant collinearity. If a continuous variable did not meet the linearity assumption, it was converted into a categorical variable based on quartiles. For all analyses, two-sided *p*-values < 0.05 were considered statistically significant.

## 3 Results

### 3.1 Population characteristics

A total of 219 eligible patients with AIS were included in the current analysis; meanwhile, 219 age- and sex-matched individuals who underwent medical examinations were included as the control group. The results showed that patients with AIS had higher baseline systolic blood pressure, diastolic blood pressure, and fasting blood glucose. LDL and HDL levels in the AIS group were significantly lower than those in the control group. The proportion of renal cysts was considerably higher among patients with AIS than among controls. No statistically significant differences were found in BMI, creatinine, triglycerides, or homocysteine between the two groups. Detailed information is displayed in Table 1. Traditional risk factors for cerebrovascular disease were recorded among patients with AIS, and 199 (90.9%) young patients with stroke had at least one risk factor, as displayed in Table 2.

**Table 1:** Baseline characteristics of young AIS patients and controls undergoing medical examinations.

Characteristic	AIS (*n* = 219)	Control (*n* = 219)	*p* value
Age (years)	45 (40–48)	44 (38–47)	0.108
Male (n, %)	181 (82.6%)	181 (82.6%)	1.000
BMI (kg/m^2^)	24.75 (22.92–27.45)	25.51 (23.29–27.69)	0.292
SBP (mmHg)	146 (131–162)	124 (115–132)	<0.001**
DBP (mmHg)	91.05 ± 17.05	79.43 ± 11.32	<0.001**
FBG (mmol/L)	5.27 (4.53–7.34)	4.77 (4.41–5.12)	<0.001**
Renal cyst (%)	52 (23.7%)	24 (11.0%)	<0.001**
Creatinine (μmol/L)	68.50 (56.75–82.25)	69.00 (60.00–78.00)	0.913
LDL (mmol/L)	3.07 (2.48–3.60)	3.23 (2.72–3.71)	0.016*
Triglycerides (mmol/L)	1.85 (1.30–2.54)	1.74 (1.11–2.53)	0.214
HDL (mmol/L)	0.94 (0.80–1.07)	1.15 (0.99–1.39)	<0.001**
Homocysteine (μmol/L)	11.20 (9.40–13.50)	11.00 (9.70–12.80)	0.494

AIS, acute ischemic stroke; BMI, body mass index; DBP, diastolic blood pressure; FBG, fasting blood glucose; HDL, high-density lipoprotein; LDL, low-density lipoprotein; SBP, systolic blood pressure. *P < 0.05, **P < 0.001.

**Table 2:** Traditional vascular risk factors in young adults with AIS.

Risk factors	Number (*n*)	Proportion (%)
Hypertension	147	67.12
Hyperlipidemia	104	47.49
Diabetes mellitus	54	24.66
Atrial fibrillation	1	0.46
Current smoking status	136	62.10
Current alcohol consumption	80	36.53
One or more risk factors	199	90.9

AIS, acute ischemic stroke.

### 3.2 The association between renal cysts and the occurrence of AIS in young adults

To explore the independent risk factors for AIS in young adults, binary logistic regression analysis was performed, and the adjusted odds ratios (aORs) and 95% confidence intervals (CIs) were calculated, as presented in Table 3 and Figure 2. In the regression model, we included all potential risk factors identified in the univariable analyses shown in Table 1, and adjusted for potential confounding factors such as age and sex. DBP was not included in the model because of collinearity with SBP. The results showed that SBP level 3 (aOR 3.717, 95% CI 1.819–7.595, *p* < 0.001), SBP level 4 (aOR 17.936, 95% CI 7.644–42.084, *p* < 0.001), FBG (aOR 1.296, 95% CI 1.115–1.506, *p* = 0.001), and renal cysts (aOR 2.980, 95% CI 1.379–6.438, *p* = 0.005) were identified as independent risk factors for AIS among young adults.

**Table 3:** Logistic regression analysis for risk factors for early-onset stroke.

Variable	OR (95% CI)	*p*-value	Adjusted OR (95% CI)	*p*-value
Age	1.031 (0.997–1.065)	0.071	0.998 (0.951–1.048)	0.946
Sex	1.000 (0.610–1.640)	1.000	0.378 (0.171–0.834)	0.016*
SBP (mmHg)		<0.001**		<0.001**
SBP_1 (≤119)	0.814 (0.452–1.464)	0.491	0.654 (0.321–1.331)	0.241
SBP_2 (120–132)	4.115 (2.324–7.284)	<0.001**	3.717 (1.819–7.595)	<0.001**
SBP_3 (≥133)	17.661 (8.675–35.957)	<0.001**	17.936 (7.644–42.084)	<0.001**
FBG (mmol/L)	1.399 (1.233–1.588)	<0.001**	1.296 (1.115–1.506)	0.001**
Renal cyst (%)	2.530 (1.495–4.281)	0.001	2.980 (1.379–6.438)	0.005**
LDL (mmol/L)		0.091		0.102
LDL_1 (≤2.60)	0.584 (0.341–0.999)	0.050	0.504 (0.234–1.085)	0.080
LDL_2 (2.61–3.17)	0.629 (0.367–1.076)	0.090	0.429 (0.198–0.932)	0.032
LDL_3 (≥3.18)	0.522 (0.304–0.895)	0.018	0.425 (0.196–0.923)	0.031
HDL (mg/dL)	0.893 (0.870–0.917)	<0.001**	0.885 (0.855–0.916)	<0.001**

^*^Data were adjusted for age and sex. FBG, fasting blood glucose; HDL, high-density lipoprotein; LDL, low-density lipoprotein; SBP, systolic blood pressure. **P < 0.001.

**Figure 2:**
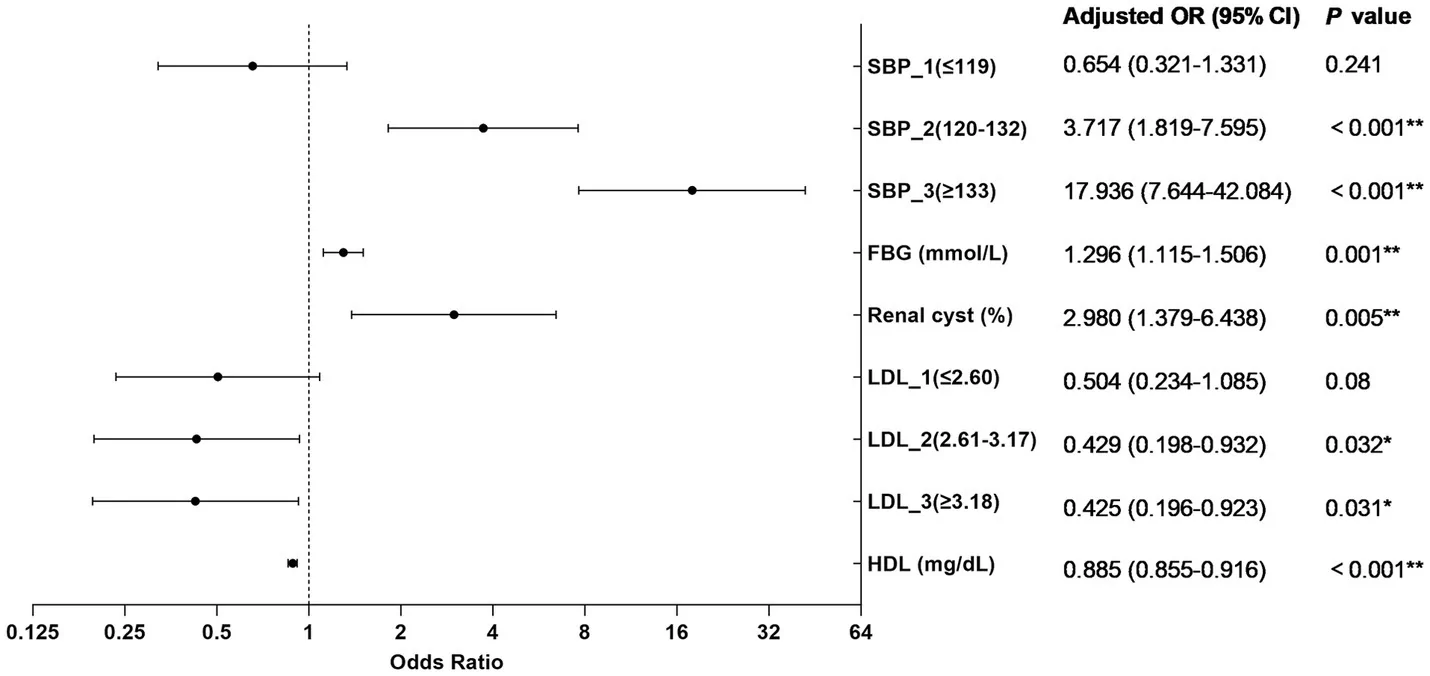
Odds ratios (ORs) and 95% confidence intervals (CIs) for risk factors for cerebral infarction in young adults with AIS. Adjusted for age and sex. FBG, fasting blood glucose; HDL, high-density lipoprotein; LDL, low-density lipoprotein; SBP, systolic blood pressure.

### 3.3 Comparison of the risk of stroke in young adults with single and multiple renal cysts

To compare the risk of stroke in young adults between multiple renal cysts (not sufficient for a diagnosis of ADPKD) and a single renal cyst, the included subjects were divided into three groups based on the number of renal cysts (no renal cyst, a single renal cyst, and multiple renal cysts), and pairwise comparisons using the chi-square test were conducted. There was a statistically significant difference between the no-renal-cyst and single-renal-cyst groups, as well as between the no-renal-cyst and multiple-renal-cyst groups; no difference was observed between multiple renal cysts and a single renal cyst, as shown in Figure 3. The results suggest that single and multiple renal cysts are equally associated with stroke in young patients.

**Figure 3:**
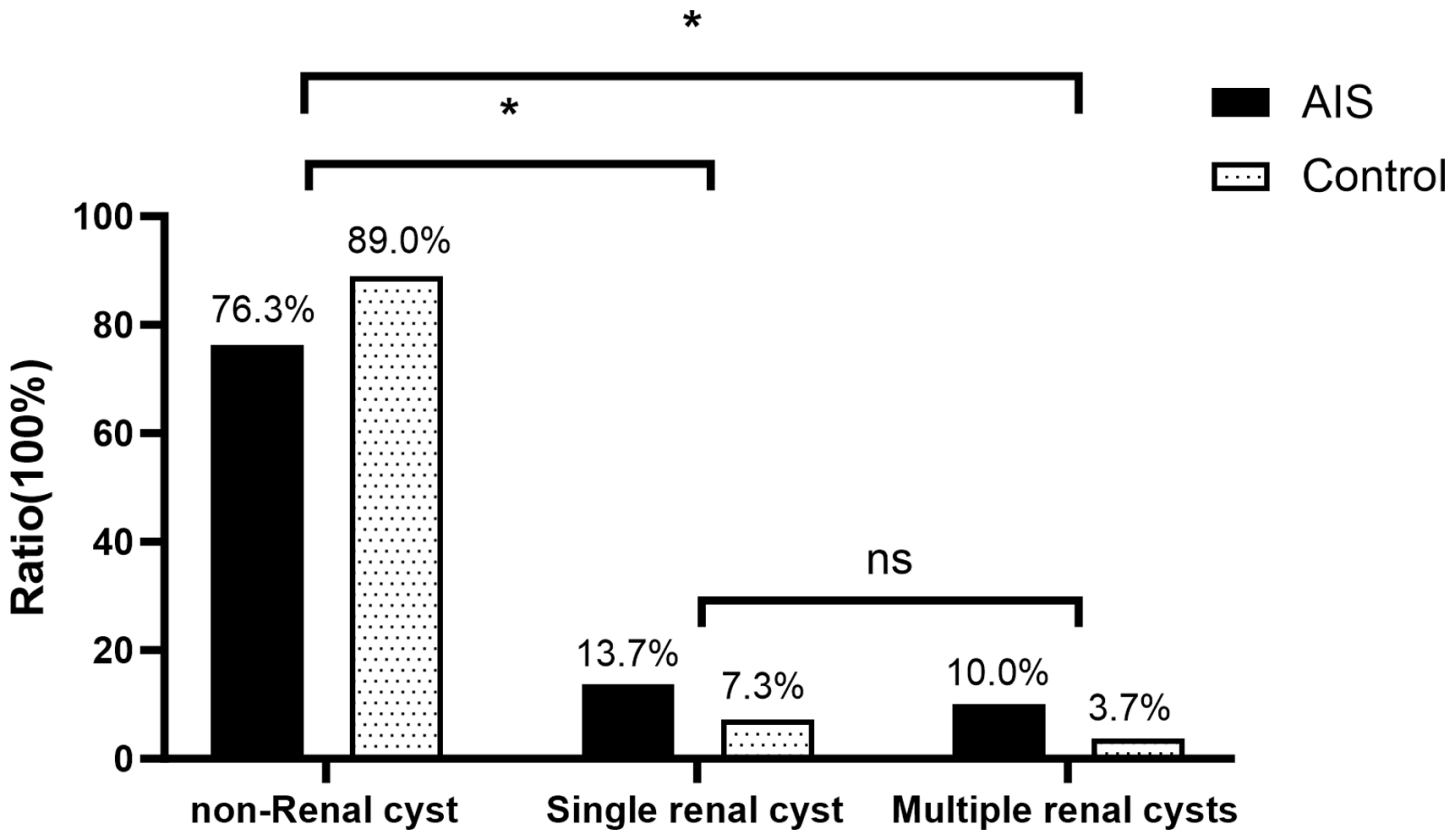
Comparison of no renal cyst, a single renal cyst, and multiple renal cysts between young adults with and without AIS. AIS, acute ischemic stroke.

### 3.4 Comparison of renal cyst features between young AIS patients and control subjects

To compare renal cyst characteristics between the stroke group and the control group, we assessed the number, size, and lateral distribution of cysts. No statistically significant differences were observed between the two groups for any of these parameters. Detailed information is displayed in Table 4.

**Table 4:** Renal cyst characteristics in young patients with acute ischemic stroke (AIS) versus controls.

Renal cyst characteristics	AIS (*n* = 52)	Control (*n* = 24)	*p*
Size (maximum diameter of renal cyst, mm)	12 (10–19.75)	14 (11.25–20)	0.388
Number of cysts			0.804
1	30 (57.7%)	15 (62.5%)	
2	22 (42.3%)	9 (37.5%)	
Laterality			0.344
Left	20 (38.5%)	11 (45.8%)	
Right	15 (28.8%)	9 (37.5%)	
Bilateral	17 (32.7%)	4 (16.7%)	

### 3.5 Subgroup analysis

The subgroup analysis was conducted in patients with AIS. Among young patients with stroke, no statistically significant differences in SBP, DBP, FBG, creatinine, LDL, HDL, or triglycerides were detected between patients with and without renal cysts, suggesting that the presence of renal cysts in this cohort of young patients with AIS was not associated with significant differences in the measured metabolic and renal parameters. Detailed information is presented in Table 5. NIHSS scores did not differ significantly between patients with and without renal cysts, suggesting that renal cysts were associated with the risk of stroke but were not associated with greater stroke severity.

**Table 5:** Comparison of characteristics between patients with renal cyst and no renal cyst among all AIS subjects.

Characteristic	Renal cyst (*n* = 52)	No renal cyst (*n* = 167)	*p* value
SBP (mmHg)	145.06 ± 22.40	146.72 ± 24.18	0.660
DBP (mmHg)	91.12 ± 15.57	91.02 ± 17.53	0.973
FBG (mmol/L)	5.24 (4.68–6.69)	5.31 (4.50–7.45)	0.659
Creatinine (μmol/L)	73.00 (60.00–88.50)	66.00 (55.00–80.00)	0.091
Proteinuria (%)	14 (26.9%)	39 (23.4%)	0.600
LDL (mmol/L)	3.31 (2.54–3.60)	3.03 (2.41–3.60)	0.516
HDL (mmol/L)	0.93 (0.83–1.08)	0.94 (0.79–1.07)	0.529
Triglycerides (mmol/L)	2.00 (1.46–2.45)	1.79 (1.25–2.69)	0.609
Admission NIHSS	3.00 (1.00–4.50)	2.00 (1.00–4.25)	0.688

AIS, acute ischemic stroke; DBP, diastolic blood pressure; FBG, fasting blood glucose; HDL, high-density lipoprotein; LDL, low-density lipoprotein; NIHSS, National Institutes of Health Stroke Scale; SBP, systolic blood pressure.

## 4 Discussion

The results of the current study show that, compared with the control group, the baseline SBP, DBP, and FBG of young adults with AIS were significantly higher, indicating that these traditional risk factors for cerebrovascular disease are still key contributors to stroke in young adults. Meanwhile, the study findings also suggest that there is a markedly higher proportion of renal cysts among patients with AIS than among the controls, and the results of the logistic regression analysis confirm the hypothesis that renal cysts are a risk factor for stroke in young adults. The impact of single or multiple renal cysts on AIS among young adults did not differ, and the presence of renal cysts was not significantly associated with AIS severity. Notably, the results indicate that the presence of renal cysts, rather than their specific morphological characteristics (size, number, or laterality), may be closely associated with the occurrence of cerebral infarction in young adults. In addition, no significant correlation was observed between the presence of renal cysts and blood pressure, glucose, or lipid levels among patients with cerebral infarction in the subgroup analysis. The LDL level was lower in the AIS group, which is inconsistent with traditional understanding. This inconsistency may be attributed to the use of lipid-lowering drugs before symptom onset in patients with AIS, as 11.0% of them were confirmed to have received lipid-lowering therapy. Regrettably, medication use in the control group was not recorded.

In our study, the median age of the included young adults with AIS was 45 years, similar to that in a previous study on stroke in young adults (43.8 years) (16). Male patients accounted for 82.6% of the sample and outnumbered the female patients. This finding is consistent with published research showing that the proportion of male patients was remarkably higher than that of female patients in the young stroke population in China, suggesting a higher proportion of men among young patients with stroke (16–18). Traditional risk factors (such as smoking, alcoholism, hypertension, diabetes, obesity, and hypercholesterolemia) were detected in approximately 80% of young patients with stroke, as reported by Aigner et al. (19). Similarly, in our study, one or more traditional risk factors were present in 199 (90.9%) young patients with AIS, suggesting that traditional cerebrovascular risk factors remain prevalent in stroke among young adults. Logistic regression analysis showed that elevated baseline blood pressure and fasting blood glucose were independently associated with stroke in young adults; this finding further emphasizes the importance of primary prevention in young adults at high risk of stroke.

Several studies have found that cardiovascular disease is a major cause of death in patients with ADPKD (20–22); because of the similar pathogenesis, the association between ADPKD and stroke has begun to attract attention. More than 20 years ago, Schievink et al. discovered that patients with ADPKD are at an increased risk of developing intracranial arterial dolichoectasia and dissections, which may cause stroke (23). Subsequent clinical observational studies have confirmed that patients with ADPKD have a higher risk of cerebral hemorrhage, subarachnoid hemorrhage, and ischemic stroke than the population without ADPKD (10, 24, 25). However, there are few reports on the relationship between simple renal cysts and stroke; a simple renal cyst is a round pouch of fluid that forms on or in the kidney.

In our current study, the results showed that renal cysts were associated with stroke in adults aged 18–50 years, indicating that renal cysts had an association with stroke similar to that observed for ADPKD. Our current study differs from the aforementioned studies in the following aspects: First, this study estimated the association between AIS and simple renal cysts (including single and multiple renal cysts), rather than ADPKD. The results suggest that both single and multiple simple renal cysts are associated with AIS. Second, previous studies on patients with stroke accompanied by ADPKD or simple renal cysts included all age groups, whereas our current study focused only on stroke in young adults, which addresses the gap in research on the correlation between renal cysts and stroke in young adults. The results indicated that renal cysts are a potential hidden risk factor for stroke in young adults, in addition to well-known risk factors such as hypertension, diabetes, and dyslipidemia, which warrants further investigation in future prospective studies. Finally, in order to further compare the increased risk of AIS among young adults with single and multiple renal cysts, we carried out a pairwise comparison and detected no statistically significant difference, indicating that there was no statistically significant difference between single and multiple renal cysts in their association with stroke.

Several aspects should be considered regarding the additional risk of AIS attributed to renal cysts. First, renal cysts are an independent risk factor for hypertension, and the prevalence of hypertension is significantly associated with renal cyst load (26–28), especially in young adults (29). Second, renal cysts seem more likely to coexist with obesity, diabetes, and metabolic syndrome (30–32), which are recognized risk factors for AIS. Hyperuricemia has also been shown to be associated with stroke occurrence, as Şengüldür and Demir reported that hyperuricemia (serum uric acid ≥ 7 mg/dL) was associated with a 2.4-fold increased risk of ischemic stroke (33). Patients with simple renal cysts exhibit significantly elevated serum uric acid levels compared with patients without cysts (34). Third, recent evidence suggests an association between renal cysts and functional damage to the renal parenchyma (35), and people with end-stage renal disease have a substantially higher risk of stroke, especially young people; patients with end-stage renal disease aged 35–39 years had an 11-fold increased risk of stroke (36). Fourth, the presence of simple renal cysts was an independent predictor of increased arterial stiffness, which is the most important pathogenic mechanism underlying ischemic stroke (37, 38). Finally, patients with renal cysts seem more likely to have abnormal development of blood vessel walls, as they are at a remarkably high risk of developing abdominal aortic aneurysms and thoracic aortic disease (39, 40).

The limitations of this study are as follows: (1) This was a retrospective study. Information on the medical history and medication use of the controls was not recorded, making it impossible to compare traditional risk factors (hypertension, diabetes, hyperlipidemia, and atrial fibrillation) and medication use (antihypertensive therapy, antiplatelet use, and statin therapy) between the two groups. Fortunately, all individuals had records of baseline blood pressure, fasting blood glucose, and lipid levels, which could reflect the status of these traditional risk factors. It should be noted that we cannot exclude the possibility that these values were influenced by acute stroke-related physiological responses or early therapeutic interventions. (2) Selection bias may have been introduced because only patients who underwent renal ultrasonography were included. Although we compared the included and excluded patients with AIS and found no significant differences in key demographic and clinical parameters, we cannot rule out the possibility that requiring ultrasound examination may have selectively excluded patients with milder or more severe strokes, potentially affecting the generalizability of our findings. (3) The lack of documentation on the use of lipid-lowering drugs prevented us from providing a reasonable explanation for the lower LDL level in the AIS group than in the control group. (4) This study was conducted at a single center in China, which limits the generalizability of the results to populations of other ethnicities.

## 5 Conclusion

In conclusion, our research indicates that renal cysts are associated with ischemic stroke in young adults and thus merit clinical awareness. Prospective multicenter cohort studies are needed to further evaluate this association and clarify potential mechanisms.

## Data Availability

The raw data supporting the conclusions of this article will be made available by the authors, without undue reservation.
