# Supplementary material for: Simple renal cysts are associated with ischemic stroke in young adults: a case–control study
Source: Front Neurol. 2026 Oct 5;17:1926737. doi: 10.3389/fneur.2026.1926737 (PMC13677531; doi:10.3389/fneur.2026.1926737)
Supplement: Supplementary file 1 [file Table_1.DOCX]

| **Supplementary Table S1 Baseline characteristics and traditional vascular risk factors of included and excluded young AIS patients** | | | |
| --- | --- | --- | --- |
| **Characteristic** | **Included AIS (n=219)** | **Excluded AIS (n=132)** | ***P* value** |
| Age (years) | 45 (40-48) | 45 (40-48) | 0.340 |
| Male (n, %) | 181(82.6%) | 106(80.30%) | 0.572 |
| BMI (kg/m^2^) | 24.75(22.92-27.45) | 25.00(23.38-28.08) | 0.356 |
| SBP (mmHg) | 146(131-162) | 141(125-156) | 0.069 |
| DBP (mmHg) | 91.05±17.05 | 88.39±15.98 | 0.148 |
| FBG (mmol/L) | 5.27(4.53-7.34) | 5.06(4.53-6.34) | 0.212 |
| Creatinine (μmol/L) | 68.50(56.75-82.25) | 65.50(54.00-79.00) | 0.511 |
| LDL (mmol/L) | 3.07(2.48-3.60) | 3.24(2.56-3.89) | 0.173 |
| Triglyceride (mmol/L) | 1.85(1.30-2.54) | 1.87(1.43-2.99) | 0.465 |
| HDL (mmol/L) | 0.94(0.80-1.07) | 0.89(0.74-1.09) | 0.162 |
| Homocysteine (μmol/L) | 11.20(9.40-13.50) | 10.7(9.3-12.4) | 0.187 |
| Admission NIHSS | 2.00(1.00-4.00) | 2.00(1.00-4.00) | 0.172 |
| Hypertension | 147(67.12%) | 81(61.36%) | 0.299 |
| Hyperlipidemia | 104(47.49%) | 71(53.79%) | 0.272 |
| Diabetes mellitus | 54(24.66%) | 42(31.82%) | 0.174 |
| Atrial fibrillation | 1(0.46%) | 0(0%) | 1.000 |
| Current smoking | 136(62.10%) | 69(52.27%) | 0.075 |
| Current drinking | 80(36.53%) | 38(28.79%) | 0.162 |
| AIS, acute ischemic stroke; BMI, body mass index;DBP, diastolic blood pressure; FBG, fasting blood glucose; HDL, high density lipoprotein; LDL, low density lipoprotein; SBP, systolic blood pressure. | | | |

| **Supplementary Table S2 Collinearity analysis of variables in logistic regression model** | | |
| --- | --- | --- |
| **Variables** | **Tolerance** | **VIF** |
| SBP | 0.873 | 1.146 |
| LDL | 0.969 | 1.031 |
| HDL | 0.912 | 1.096 |
| FBG | 0.950 | 1.053 |
| Renal cyst | 0.994 | 1.006 |
| DBP, diastolic blood pressure; FBG, fasting blood glucose; HDL, high density lipoprotein; LDL, low density lipoprotein; SBP, systolic blood pressure; VIF, variance inflation factor. | | |
